# Emerging Therapeutic Approaches to Pancreatic Adenocarcinoma: Advances and Future Directions

**DOI:** 10.1007/s11864-025-01352-2

**Published:** 2025-08-30

**Authors:** Chengwei Peng, Paul E. Oberstein

**Affiliations:** 1 Department of Hematology/Oncology, Feinberg School of Medicine, Northwestern University, Chicago, IL USA; 2 Department of Hematology/Oncology, NYU Langone Health, New York University, New York, NY USA

**Keywords:** Pancreatic adenocarcinoma, KRAS inhibition, Claudin 18.2, Clinical trials

## Abstract

Pancreatic adenocarcinoma remains a leading cause of cancer-related mortality worldwide. Although surgery can be curable for a subset of patients, the five-year overall survival remains less than 15%. Despite extensive molecular characterization of pancreatic cancer, cytotoxic chemotherapy has served as the major component in therapeutic management. A major driver of pancreatic adenocarcinoma is mutations in KRAS, present in over 90% of cases. However, attempts to inhibit KRAS through upstream and downstream targets through the mitogen-activated protein kinase pathway have not been successful in the past. Despite this, multiple KRAS inhibitors have recently entered clinical trials and have shown promising results. These inhibitors have the potential to dramatically alter the landscape of treatment. In parallel, immunological approaches utilizing vaccines and bispecific antibodies are also in clinical development. Given these rapid new developments, the future of pancreatic cancer treatment will likely be determined by discovering the appropriate combinations of targeted and immune-based treatments.

## Introduction

Pancreatic cancer is currently the 7th most common cancer globally and 4th most common cause of cancer mortality worldwide [1]. In the United States, pancreatic cancer is estimated to become the second leading cause of cancer mortality after lung cancer by 2030[2]. Despite an increase in 5-year survival from 5 to 13% in the last 10 years, the overall mortality remains high [3, 4].

**Fig. 1:**
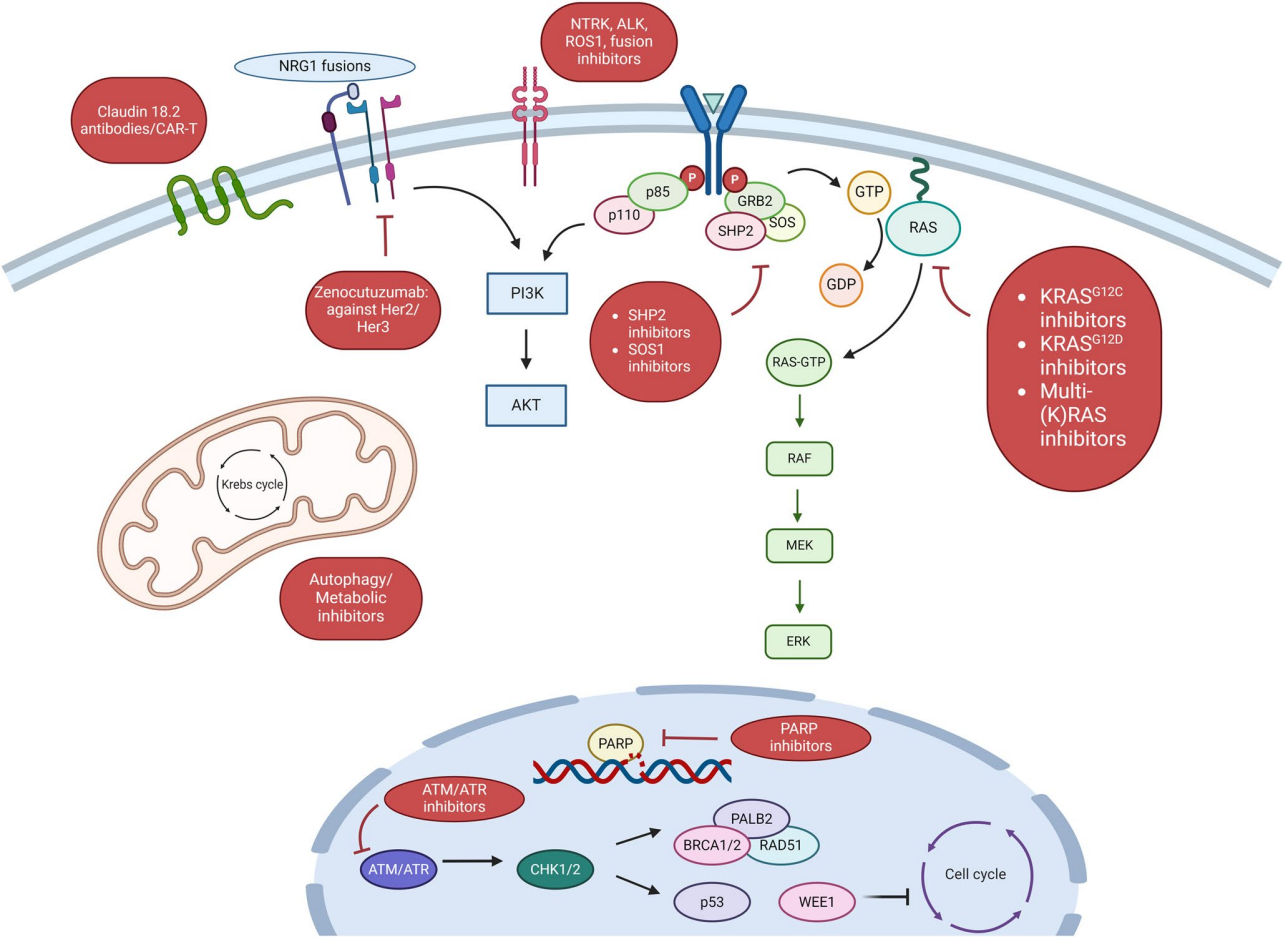
Major pathways targeted by novel treatment options for pancreatic adenocarcinoma. Created with BioRender

Among pancreatic cancers, pancreatic ductal adenocarcinoma (PDAC) comprises the majority of cases (~ 85–90%) and we will primarily focus on this subtype in our discussion [5, 6]. Although survival remains low in advanced disease, the development of *KRAS* inhibitors and immunotherapeutic approaches potentially serve as major advances in the treatment of PDAC. In this review, we will highlight recent novel therapeutic approaches to improving survival in PDAC (Fig. 1).

## Challenges in Current Management

Currently, cytotoxic chemotherapy plays a key role across all stages of PDAC. A list of key phase III trials is included in Table 1 [7–14]. Despite intensification of chemotherapy regimens with gemcitabine/*nab*-paclitaxel (G/A) and modified FOLFIRINOX/NALIRIFOX (5-FU, oxaliplatin, irinotecan/liposomal irinotecan), PDAC remains an incurable disease unless surgical resection is achievable. However, localized disease comprises less than 30% of patients, not all of whom will have potentially resectable disease [15]. In unresectable, locally advanced disease, use of chemotherapy and radiation have limited efficacy in conversion to resectability, with the proportion of patients who subsequently qualify for resection less than 10%[16].

**Table 1:** Phase III clinical trials in PDAC

Trial	Clinical setting	Treatment (number of patients)	Results
PREOPANC [7]	Neoadjuvant, resectable and borderline resectable	Gemcitabine plus radiation (119) versus Gemcitabine (127)	Primary endpoint mOS: 15.7 months versus 14.3 months (HR = 0.73; 95% CI, 0.56–0.96; *p* = 0.025) *In subgroup analysis, p-value was not significant in the resectable group
PRODIGE 24/CCTG PA6 [8]	Adjuvant	Modified FOLFIRINOX (247) versus Gemcitabine (246)	Primary endpoint mDFS: 21.6 months versus 12.8 (HR = 0.58; 95% CI, 0.46–0.73; *p* < 0.001 mOS: 54.4 months versus 35.0 months (HR = 0.64; 95% CI, 0.48–0.86; *p* = 0.003).
ESPAC-4 [9]	Adjuvant	Gemcitabine/capecitabine (364) versus Gemcitabine (366)	Primary endpoint mOS: 28.0 months versus 25·5 months (HR = 0.82, 95% CI 0.68–0.98, *p* = 0.032).
PRODIGE 4/ACCORD 11 [10]	First-line metastatic	FOLFIRINOX (171) versus gemcitabine (171)	Primary endpoint mOS: 11.1 months versus 6.8 months (HR = 0.57; 95% CI, 0.45–0.73; *p* < 0.001)
MPACT [11]	First-line metastatic	Gemcitabine/nab-paclitaxel (431) versus Gemcitabine (430)	Primary endpoint mOS: 8.5 months versus 6.7 months (HR = 0.72; 95% CI 0.62–0.83; *p* < 0.001)
NAPOLI-3 [12]	First-line metastatic	NALIRIFOX (383) versus Gemcitabine/nab-paclitaxel (387)	Primary endpoint mOS: 11.1 months versus 9.2 months (HR = 0.83; 95% CI 0.70–0.99; *p* = 0.036)
POLO [13]	First-line metastatic (maintenance)	Olaparib (92) versus placebo (62)	Primary endpoint mPFS: 7.4 months versus 3.8 months (HR = 0.53; 95% CI 0.35–0.82; *p* = 0.004) mOS: 18.9 months versus 18.1 months (HR = 0.91; 95% CI, 0.56–1.46; *p* = 0.68)
NAPOLI-1 [14]	Second-line metastatic, prior gemcitabine treatment	NALIRI (117), liposomal irinotecan (151), versus 5-FU/folinic acid (149)	Primary endpoint mOS: NALIRI versus 5-FU/folinic acid: 6.1 months versus 4.2 months (HR = 0.67; 95% CI, 0.49–0.92; *p* = 0.012) Liposomal irinotecan versus 5-FU/folinic acid: 4.9 months versus 4.2 months (HR = 0.99; 95% CI, 0.77–1.28; *p* = 0.94)

Several factors have been proposed to explain the challenges of treatment. At a biological level, pancreatic cancer is often a systemic disease even in localized stages. Thus, despite surgery, recurrence rates remain high [8, 17]. The tumor microenvironment of PDAC within the pancreas has been characterized by a distinctive fibrotic stroma which has been hypothesized to limited drug delivery and infiltration by CD8 + T-cells[18, 19]. Clinical complications such as cholangitis from stents, cachexia, and malignant ascites also add to complexity in management [20–22]. Despite the use of intensive multi-agent chemotherapy regimens, overall response rates (ORR) and survival (OS) remain low, indicating that new approaches are necessary.

## Targeted Therapy Based on Genomic Mutations

Improvements in sequencing technology have led to a deeper understanding of all cancers, including PDAC. Large genomic analyses of PDAC have consistently shown alterations in *KRAS* (> 90%), *TP53* (~ 70–80%), *CDKN2A* (~ 10–40%), and *SMAD4* (~ 20–40%)[23–28]. Given the high prevalence of mutations in KRAS, targeting KRAS and its regulators and effectors has long been a goal in treatment of pancreatic cancer. However, direct KRAS inhibition and targeted therapies have not been successful in PDAC until the last few years (Fig. 1).

Genomic studies using transcriptomics have also identified distinct subtypes of PDAC, most commonly divided into classical and basal subtypes [29]. Based on current data, these subtypes appear to have different prognosis and responses to chemotherapy [30]. Based on the COMPASS trial evaluating first-line chemotherapy in the metastatic setting, the median OS (mOS) for classical subtype was 10.0 months (95% CI, 7.5–14.9) compared to 6.3 months (95% CI, 4.4–not reached) for basal subtype (HR 0.39; 95% CI, 0.18–0.86; *p* = 0.02). In addition, median progression-free survival (mPFS) was significantly different among classical versus basal subtype, mPFS of 8.5 months (95% CI, 6.5-not reached) compared to PFS of 2.7 months (95% CI, 2.1-not reached) respectively. Despite these compelling results, molecular subtyping of pancreatic cancer has not yet been translated into actionable treatment strategies. Given the targeted regimens we will discuss here, prediction of response based on molecular subtypes will continue to be an important question.

### KRAS-Mutated PDAC

The MAPK (mitogen-activated protein kinase) pathway represents a key signaling pathway in cancer biology [31, 32]. Within this pathway, significant mutations have been found in RAS proteins (isoforms *KRAS*,* NRAS*,* HRAS*). Historically, targeting mutated RAS proteins has been difficult due to the lack of binding sites on the protein’s smooth surface. However, discovery of a new allosteric pocket in KRAS G12C mutants heralded the development of covalent small molecules that bind to KRAS^G12C^ in its GDP-bound (inactivated) state [33].

KRAS G12C inhibitors were first developed clinically in NSCLC and two are currently FDA approved: sotorasib and adagrasib. In pancreatic cancer, sotorasib demonstrated an ORR of 21% despite 79% of patients having already received at least two lines of therapy [34]. In the phase II trial KRYSTAL-1, adagrasib, 5 of 10 patients (50%) had partial responses while the remaining 5 had stable disease, showing a promising overall disease control rate of 100%[35]. Due to these results, treatment with KRAS G12C inhibitors are now listed as an option in NCCN guidelines for pancreatic cancer [36]. Multiple alternative KRAS G12C inhibitors are in development including those that target the GTP-bound (activated) state such as RMC-6291 (NCT05462717) (Table 2) [37, 38].

**Table 2:** Inhibitors of RAS

Mechanism	Drug	Phase of study	NCT	Details	Status
KRAS^G12C^ inhibitor – GDP state	Sotorasib	Approved for NSCLC, phase I/II follow-up for PDAC	NCT03600883	In PDAC (*n* = 38), ORR 21%; mPFS 4.0 months (95% CI, 2.8 to 5.6), mOS 6.9 months (95% CI, 5.0 to 9.1) [34]	Ongoing phase I/II trials with combination treatments with other inhibitors against MAPK pathway and immunotherapy agents in solid tumors (NCT04185883, NCT05638295)
	Adagrasib	Approved for advanced NSCLC, phase I/II follow-up for PDAC	NCT03785249	In PDAC (*n* = 21), ORR 33%; mPFS 5.4 months (95% CI, 3.9 to 8.2), mOS 8.0 months (95% CI, 5.2 to 11.8) [35]	Ongoing phase I trials with combination treatments with other MAPK pathway and immunotherapy agents in solid tumors (NCT05178888, NCT04330664, NCT04613596)
	Divarasib (GDC-6036)	I/II	NCT04449874	In PDAC: 3/7 PR [37]	Ongoing studies in combination with SHP2 (GDC-1971)
	Glecirasib (JAB-21822)	I/II	NCT05002270, NCT05009329	In PDAC (*n* = 28), ORR 46.4%; mPFS 5.5 months (95% CI, 1.2 to 13.1) [38]	Ongoing phase II in PDAC, combination with SHP2 (JAB-3312) (NCT06008288, NCT05288205)
	Other agents in clinical trials: LY3537982 (NCT04956640), MK-1084 (NCT05067283), IBI351 (NCT05005234), D3S-001 (NCT05410145), HBI-2438 (NCT05485974)				
KRAS^G12C^ inhibitor – GTP state	RMC-6291	I	NCT05462717	Monotherapy, also in combination with pan-RAS inhibitor RMC-6236 NCT06128551	Recruiting
	FMC-376 *targets both GDP and GTP KRAS^G12C^	I/II	NCT06244771	Monotherapy	Recruiting
	BBO-8520 *targets both GDP and GTP KRAS^G12C^	I	NCT06343402	Currently only in NSCLC as monotherapy and in combination with anti-PD1	Recruiting
KRAS^G12D^ inhibition	RMC-9805 *inhibitor targeting GTP bound state	I	NCT06040541	ORR 30% and DCR of 80% in PDAC patients [39]	Planned for combination with pan-RAS RMC-6236 and other chemotherapy/targeted agents in NCT06445062
	HRS-4642 *inhibitor targeting both GDP and GTP KRAS^G12D^	I	NCT05533463	Response in 1/18 patients (only 1 PDAC in cohort) [40]	Planned for combination treatment with chemotherapy, nimotuzumab (EGFR antibody) and adebelimab (anti-PD1) NCT06427239, NCT06587061, NCT06770452, NCT06770452
	ASP3082 KRAS^G12D^ degrader	I	NCT05382559	3/7 showing PR in PDAC [41]	Recruiting
	Other agents in clinical trials: MRTX1133 (NCT05737706), GFH375 (NCT06500676), TSN1611 (NCT06385925), INCB161734 (NCT06179160), QTX3046 (NCT06428500), QTX 3034 *KRAS^G12D^ preferring multi-KRAS inhibitor (NCT06227377), LY3962673 (NCT06586515), QLC1101 (NCT06403735), LY3962673 (NCT06586515), AZD0022 (NCT06599502)				
KRAS^G12V^ inhibition, *see also Vaccine Table for other therapeutics*	QTX 3544 *KRAS^G12V^ preferring multi-KRAS inhibitor	I	NCT06715124	Monotherapy and in combination with cetuximab	Recruiting
	siLODER system against KRAS G12V/D	I/II	NCT01676259	In combination with chemotherapy, ORR = 56% [42]	Similar agent (SIL-204) planned for phase II/III trial
Mulit-KRAS inhibition	PF-07934040	I	NCT06447662	Monotherapy and in combination with chemotherapy (G/A)	Recruiting
	PF-07985045	I	NCT06704724	Monotherapy and planned combination with chemotherapy (G/A) and other targeted agents including SHP2 (PF-07284892)	Recruiting
	LY4066434	I	NCT06607185	Monotherapy and planned combination with chemotherapy (G/A and mFOLFIRINOX)	Recruiting
	BGB-53,038	I	NCT06585488	Monotherapy and combinations planned	Recruiting
	BI 3,706,674	I	NCT06056024	Currently being studied in gastric and esophageal cancers	Recruiting
Pan-RAS Inhibition	RMC-6236	I/II, III	NCT05379985 NCT06445062 NCT06625320	In phase I PDAC (*n* = 42) with KRAS G12 mutations, ORR 29%; mPFS 8.5 months (95% CI, 5.3 to 11.7), mOS 14.5 months (95% CI, 8.8 to NE) [43]	Ongoing phase III as monotherapy versus chemotherapy in 2nd line PDAC, phase I/II for combination treatments
	RSC-1255	I	NCT04678648	Monotherapy	Recruiting

Although KRAS G12C inhibitors represent a significant advancement in therapeutics, in pancreatic cancer, KRAS G12C mutation has a frequency of 1–2%, indicating targeting of alternative *KRAS* mutations is necessary. However, not all pharmacological properties of KRAS G12C inhibitors can be easily transferred to other allelic mutations. A fairly unique property of KRAS G12C is that it spends sufficient time in the GDP state to be inhibited by sotorasib and adagrasib, both which target the inactive GDP-bound conformation [44]. In addition, the cysteine change in protein structure also allows for covalent inhibition, which has not been feasible with other allelic mutations. Despite these structural challenges, both KRAS mutant specific and pan-RAS/KRAS inhibitors are in clinical development.

Furthest in clinical development is a pan-RAS inhibitor, RMC-6236 (Daraxonrasib). Mechanistically, RMC-6236 binds the active GTP-bound form of RAS in conjunction with chaperone protein cyclophilin A to create a tricomplex compound with RAS [45]. In phase I trial in PDAC, ORR was 29% and DCR was 91% among KRAS G12 mutations in second-line therapy [43]. mPFS in the second line was 8.5 months and mOS was 14.5 months, comparing favorably to historical data (mPFS of 3.1 months and mOS of 6.1 months with NALIRI) [14]. Based on these data, a phase III trial (RASolute 302) evaluating RMC-6236 versus chemotherapy in second-line PDAC has been initiated. Multi-KRAS targeting inhibitors are also currently in clinical evaluation (Table 2).

Mutation specific inhibitors against KRAS G12D, the most frequent KRAS mutation in PDAC, are also in clinical development. RMC-9805 is a KRAS G12D inhibitor that targets the GTP-bound state through a similar mechanism to RMC-6236. Results from its phase I/Ib trial demonstrated a response rate of 30% and DCR of 80% in PDAC patients [39]. Further exploration as monotherapy and in combination with RMC-6236 are planned. Clinical data has also been reported for HRS-4642, which binds to both the GDP and GTP forms of KRAS G12D [46]. However only 1 out of 18 patients had a PR (enrollment of PDAC was limited to 1 patient) [40]. Another KRAS G12D inhibitor is MRTX1133, a non-covalent inhibitor that targets the GDP-bound state of KRAS G12D [47, 48]. In immune competent genetically engineered mouse models of PDAC (with murine Kras G12D and Trp53 mutations), MRTX1133 treatment resulted in near complete responses at two weeks [49]. The effect of MRTX1133 appeared to be partially dependent on CD8 + T-cells, indicating potential synergy with immunotherapy (see below) [50]. MRTX1133 has recently entered into phase I/II clinical trial (NCT05737706). In addition to small molecule inhibitors, KRAS^G12D^ degraders have also been developed. ASP3082 which utilizes a E3 ligase system for proteolysis is in active phase I clinical trials [51]. From the dose-escalation phase, ORR was 33.3% among all tumors, with 3/7 showing PR in PDAC at the highest dose level reported [41]. Inhibitors to KRAS G12V, the second most common *KRAS* mutation in PDAC, are also being developed (Table 2) [52].

Other strategies targeting KRAS in pancreatic cancer include KRAS directed vaccines (see immunotherapy section below) and locally delivered eluting devices targeting G12D mutation. A biodegradable implantable device (LODER) that uses RNA inhibition of KRAS G12D and KRAS G12V showed preclinical activity in mouse models [53]. A phase II trial with 48 patients with locally advanced PDAC utilizing the siG12D/V-LODER system in combination with chemotherapy resulted in an ORR of 56% and 67% improvement in resectability [42]. A next-generation version of this system is being planned for future trials. In addition, broader siRNA approaches outside of physical devices are also in development for KRAS. Use of a special nanoparticle delivery system through exosomes to deliver siRNA is also currently being studied [54].

Although the ability of KRAS inhibition to change clinical outcomes is promising for a majority of PDAC patients, clinical trials in lung and colorectal cancers using KRAS G12C inhibitors inevitably show resistance with continued treatment. The reported mechanisms of resistance are heterogenous, including new mutations in KRAS and downstream activations of the MAPK pathway as well as activations of alternative pathways such as AKT [55–57]. In PDAC, similar mechanisms have been identified from post-treatment and preclinical models, with amplifications in *KRAS*, *MYC*, *CDK6*, and *EGFR*. Of note, the basal subtype of PDAC appears to be more sensitive to KRAS inhibitors, indicating that specific targeting of subtypes may help combat resistance. Upstream combinations with SHP2 and SOS1 may also be effective (Table 3) [58, 59].

**Table 3:** Inhibitors of MAPK pathway

Mechanism	Drug	Phase	NCT	Details	Status
SHP2 inhibitor	RMC-4630	I	NCT04185883 NCT04916236 (terminated)	In combination with sotorasib, no PDAC data reported; in NSCLC (3/4) 75% PR in combination with sotorasib[58]; in combination with ERK inhibition (NCT04916236) ORR was 0% in multi-cancer study[59]	Ongoing as part of CodeBreak101 trial (NCT04185883)
	HBI-2376	I	NCT05163028	Monotherapy	Recruiting
	MK-0472	I	NCT05853367	Montherapy and in combination with KRAS^G12C^ and PD-1	Recruiting
	ET0038	I	NCT05354843	Monotherapy	Recruiting
SOS1 inhibitor	MRTX0902	I/II	NCT05578092	Monotherapy and in combination with adagrasib	Recruiting
	BAY3498264	I	NCT06659341	In combination with sotorasib	Recruiting
Downstream MAPK pathway	Ulixertinib: ERK inhibitor	I/II	NCT02608229	In combination with G/A in PDAC (*n* = 5), ORR of 20% however high percentage of AEs[60]	Other trial ongoing with ulixertinib: NCT03454035 (in combination with palbociclib)
	Avutometinib: RAF/MEK clamp	I/II	NCT05669482	In combination with G/A and defactinib; In PDAC (*n* = 8), ORR 75%[61]	Recruiting
	IMM-1-104: MEK inhibitor	I/II	NCT05585320	In combination with G/A in PDAC (*n* = 5), ORR 40%[62]	Recruiting
	IMM-6-415: MEK inhibitor	I/II	NCT06208124	Monotherapy	Recruiting
	DCC-3084: RAF inhibitor	I/II	NCT06287463	Monotherapy	Recruiting
	IPN01194: ERK inhibitor	I/II	NCT06305247	Monotherapy	Recruiting
	Other agents in trials: IK-595 (MEK/RAF molecular glue, NCT06270082); ERAS-007 (ERK inhibitor, NCT05039177); ERAS-254 (RAF inhibitor, NCT05907304);				

Finally, inhibition of KRAS^G12C^ has been associated with a proinflammatory tumor microenvironment, leading to potential synergy with immune checkpoint inhibitors [63]. While clinical trials are ongoing with available KRAS^G12C^ inhibitors and PD-1, a recent report of sotorasib with pembrolizumab or atezolizumab in NSCLC was concerning for the high rate of grade 3–4 liver toxicity of roughly 40% [64]. ORR across all cohorts was 29%. Hepatotoxicity appeared lower when adagrasib was combined with pembrolizumab in a phase Ib/II trial which showed grade 3 liver toxicity of 8–9%[65]. Whether combination treatment might benefit pancreatic cancer patients with KRAS G12C is unclear at this time. In preclinical studies of the KRAS G12D inhibitor, MRTX1133, in vivo data also shows increased CD8 + T-cells in the tumor microenvironment with treatment with increased FAS expression on cancer cells helping to facilitate T-cell cytotoxicity [50]. In addition to considering combination with checkpoint blockade, there is emerging evidence that KRAS G12C inhibitors themselves can generate new particles that are loaded onto MHC class I and can then be targeted by surface antibodies [66, 67].Whether this approach can be applied to other KRAS inhibitor classes has not been shown.

### Non-KRAS Mutated PDAC

In PDAC that do not harbor *KRAS* mutations, several studies have identified recurrent alternative alterations, many of which are targetable [68]. These include alternative MAPK pathway activation, gene fusions, and microsatellite unstable tumors (see immune section below) [69].

Gene fusions in various genes including: *NTRK*,* NRG1*,* ROS1*,* ALK*,* BRAF*,* RET*,* MET* and *FGFR2/3* have been reported in pancreatic cancer and can serve as therapeutic targets, highlighting the importance of testing for fusions in *KRAS* wildtype tumors [70, 71]. Recently, zenocutuzumab has received FDA approval for treatment of PDAC with *NRG1* fusions [72]. *NRG1* was previously identified as a novel oncogenic fusion in pancreatic cancer, serving as a fusion partner for several genes including *CD74*,* ATP1B1*,* CDH1*, and *VTCN1*. *NRG1* binds with *ERBB3*, which heterodimerizes with *ERBB2* to activate downstream pathways [71]. Zenocutuzumab is a bispecific antibody against *ERBB2/ERBB3* fusions in NRG1 + cancers. In a phase II trial, ORR was 42% in 36 patients and mPFS was 9.2 months [73]. Other fusions such as *NTRK* and *ROS1* in PDAC respond similarly to entrectinib and larotrectinib as other cancer subtypes although the data are limited to case reports given the low prevalence of these alterations [74, 75]. Responses of *MET* and *ALK* fusions to inhibitors such as crizotinib in pancreatic cancer have also been reported [70, 76]. Given these results, identification of fusions in patients using both RNA and DNA sequencing will likely be necessary.

In addition to gene fusions, MAPK pathway alterations remain a key signaling pathway even in non-*KRAS* mutated cancers. Supporting this observation, a recent phase III trial evaluated the addition of nimotuzumab, an EGFR antibody, to gemcitabine in non-KRAS mutated cancers demonstrated improvement over gemcitabine alone in terms of overall survival (18.05 versus 11.14 months, *p* = 0.036 using RMST model) [77]. The highest alterations aside from *KRAS* in the MAPK pathway are in *BRAF* mutations (and fusions), making up roughly 10–17%[68, 78, 79]. *BRAF* and *KRAS* mutations are nearly mutually exclusive across cancer types. BRAF V600E mutations are targeted successfully in melanoma with BRAF and MEK inhibitors [80]. In pancreatic cancers, small reports have indicated a potential clinical signal with BRAF and MEK inhibition [81–83]. BRAF and MEK inhibition with dabrafenib and trametinib has been approved agnostically for all tumors harboring BRAF V600E mutations, although the overall efficacy in PDAC is still unknown. Clinical trials targeting downstream proteins in the MAPK pathway such as MEK and ERK using novel agents are in process and may have efficacy in non-KRAS mutated tumors (Table 3) [60–62].

### DNA Damage and Homologous Repair Pathway

Recent studies have shown that the clinical prevalence of patients with mutations in DNA repair pathway is roughly 13–17% in PDAC [84, 85]. Although there is uncertainty as to what counts as defined mutations in this pathway, initial studies focused on germline mutations in *BRCA1/2*, which lead to DNA homologous recombination deficiency (HRD). Retrospective studies of PDAC patients with these mutations showed increased sensitivity to platinum agents, presumably due to increased susceptibility to DNA damage [86–89]. This population has been targeted in clinical trials with PARP inhibitors based on the concept of synthetic lethality (targeting of both single stranded and double stranded DNA repair), initially in ovarian cancer, before expanding to other cancer subtypes [90, 91].

In DAC, the POLO trial was an international phase III trial evaluating PARP inhibition with Olaparib. In metastatic PDAC patients with germline *BRCA1/2* mutations, maintenance PARP inhibition with Olaparib (after a minimum 16 weeks without progression on platinum-based chemotherapy), showed improvement in PFS when compared with placebo (7.4 months vs. 3.8 months; HR = 0.53; 95% CI 0.35 to 0.82; *p* = 0.004). However, there was no difference in mOS in the study (18.9 months vs. 18.1 months; HR = 0.91; 95% CI, 0.56 to 1.46; *p* = 0.68), raising the question if PARP inhibition provides significant benefit to justify potential toxicities and cost [13, 92]. In a phase II trial utilizing rucaparib as maintenance therapy after platinum-based chemotherapy in both *BRCA1/2* and *PALB2* mutated patients with advanced PDAC, PFS was 13.1 months (95% CI, 4.4 to 21.8) among 46 patients [93]. This improved PFS compared to the POLO trial may be due in part from the inclusion of LAPC patients.

Related clinical trials have focused on PARP inhibition with chemotherapy and immunotherapy (including as adjuvant therapy) in patients with *BRCA1/2* and *PALB2* mutations (both somatic and germline). Reported results so far have been mixed in these combination treatments. In a phase II trial combining the PARP inhibitor velparib with FOLFIRI in second-line treatment in all patients regardless of HRD mutations, combination therapy did not improve survival compared to FOLFIRI alone [94]. However, only 12 patients in the combination group had HRD, although they did not have improved survival compared to those who did not have HRD, 7.4 months vs. 5.1 months (HR 1.78, 95% CI 0.89–3.55, *p* = 0.10).

In terms of immunotherapy and PARP inhibition, a phase Ib/II trial compared niraparib plus nivolumab (46 patients) or niraparib plus ipilimumab (45 patients) as maintenance therapy in patients who had not progressed after 16 weeks of platinum-based chemotherapy [95]. No stratification or presence of HRD mutations were required, although both arms ultimately had the same number of patients with *BRCA1/2* and *PALB2* mutations (7 in each arm). In this trial, niraparib plus ipilimumab had a PFS at 6 months of 59.6% vs. 20.6% in the niraparib plus nivolumab combination. Whether these results are due to patient factors or real biological changes is unclear. An additional study, the phase II POLAR trial, evaluated maintenance olaparib with pembrolizumab within 3 different cohorts, A: germline *BRCA1/2* and *PALB2* with platinum response over 4 months, B: other HRD genes with platinum response over 4 months, and C: non-HRD but response on platinum for over 6 months. DCR at 6 months was 90% (26/29) in cohort A, 47% (7/15) in cohort B, and 20% (3/15) in cohort C [96]. A randomized phase II trial of olaparib and pembrolizumab is currently in progress for those with germline *BRCA1/2* mutations [97]. Of interest, a recent single institution case series reported on 12 patients with pancreatic (10 patients) and biliary cancer and HRD germline variants who were treated with combination ipilimumab and nivolumab [98]. The overall response rate was 42% with 2 PDAC patients achieving complete responses that were durable for over 24 months.

Outside of *BRCA1/2* and *PALB2* mutations, there is significant controversy on what counts as HRD. Current mutations reported in the literature include *ATM*,* ATR*,* CHEK2*,* RAD51*, and *FANC* genes. However, not all mutations predict response to PARP inhibition, similar to what has been shown in prostate cancer. Specifically, *ATM* and *CHEK2* mutations did not demonstrate an HRD signature in a recent study [99]. It is likely that tumor extrinsic factors such as the surrounding microenvironment plays a role as well, in addition to concurrent mutations [100].

Novel approaches to targeting HRD currently include inhibition of other key players within the DNA damage pathway. Among HRD related genes in pancreatic cancer, the highest in prevalence is *ATM* (2–5%). Although this mutation has not been consistently shown to respond to PARP inhibitors, preclinical testing has shown increased susceptibility to combined ATR inhibition with PARP inhibitors and chemotherapy [101, 102]. ATR plays a role in single stranded DNA repair while ATM acts on double stranded breaks, suggesting ATR inhibition can sensitize tumors to DNA damage. Combinations of ATR and PARP inhibitors trials are currently ongoing (Table 4). Selective inhibitors of PARP1 have been developed to minimize toxicity of inhibiting both PARP1 and PARP2[103]. Other targets currently undergoing early phase clinical trials include DNA polymerase theta, PARG, and tankyrase (Table 4). Whether these agents will have enough of a therapeutic window either as monotherapy or in combination will be important.

**Table 4:** Inhibitors of DNA repair pathway

Mechanism	Drug	Phase	NCT	Details	Status
PARP inhibitor	Olaparib	II	NCT04858334	Maintenance therapy for PDAC in adjuvant setting	Recruiting
		Randomized phase II	NCT04548752	Metastatic or locally advanced PDAC with BRCA1/2 mutation with stable disease after 16 weeks of standard platinum-based chemotherapy	Recruiting, Olaparib with pembrolizumab vs. Olaparib alone
		II	NCT05286827	In pancreatic acinar histology	Recruiting
	Niraparib	I	NCT04673448	In combination with dostarlimab	Recruiting
		II	NCT06747845	In combination with Ipilimumab in the maintenance setting	Not yet recruiting
PARP1 inhibitor	AZD5305	I/II	NCT04644068	Single agent and in combination cohorts	Recruiting
	VB15010	I/II	NCT06819215	Single agent	Recruiting
Dual PARP and microtubule inhibitor	AMXI-5001	I/II	NCT04503265	Monotherapy	Recruiting
PARG inhibitor	IDE161	I	NCT05787587	Monotherapy	Recruiting
Dual Inhibitor of PARP/Tankyrase	JPI-547	I	NCT05257993	In combination with chemotherapy G/A or FOLFIRINOX	Recruiting
DNA polymerase theta inhibitor	MOMA-313	I	NCT06545942	Monotherapy and in combination with olaparib	Recruiting
ATR inhibitor	RP-3500 (Camonsertib)	I	NCT04497116	Monotherapy and in combination with G/A or talazoparib	Recruiting
		I/II	NCT04972110	In combination with niraparib or olaparib	Recruiting
	Ceralasertib	II	NCT03682289	ATM loss with PDAC cohort; Monotherapy and in combination with Olaparib or durvalumab	Recruiting
	M1774	I	NCT04170153	Monotherapy and in combination with niraparib	Recruiting
		I	NCT05396833	In combination with ATM inhibitor (M4076) or anti-PD-L1 (avelumab)	Recruiting
		I	NCT05687136	In combination with DNA-PK inhibitor (M3814)	Recruiting
		I	NCT06421935	In combination with PARP1 inhibitor M9466	Recruiting
	Other ATR inhibitors: ATG-018 (NCT05338346), ART0380 (NCT05798611), IMP9064 (NCT05269316), ATRN-119 (NCT04905914)				

## Tumor and Immune Microenvironment of PDAC

**Fig. 2:**
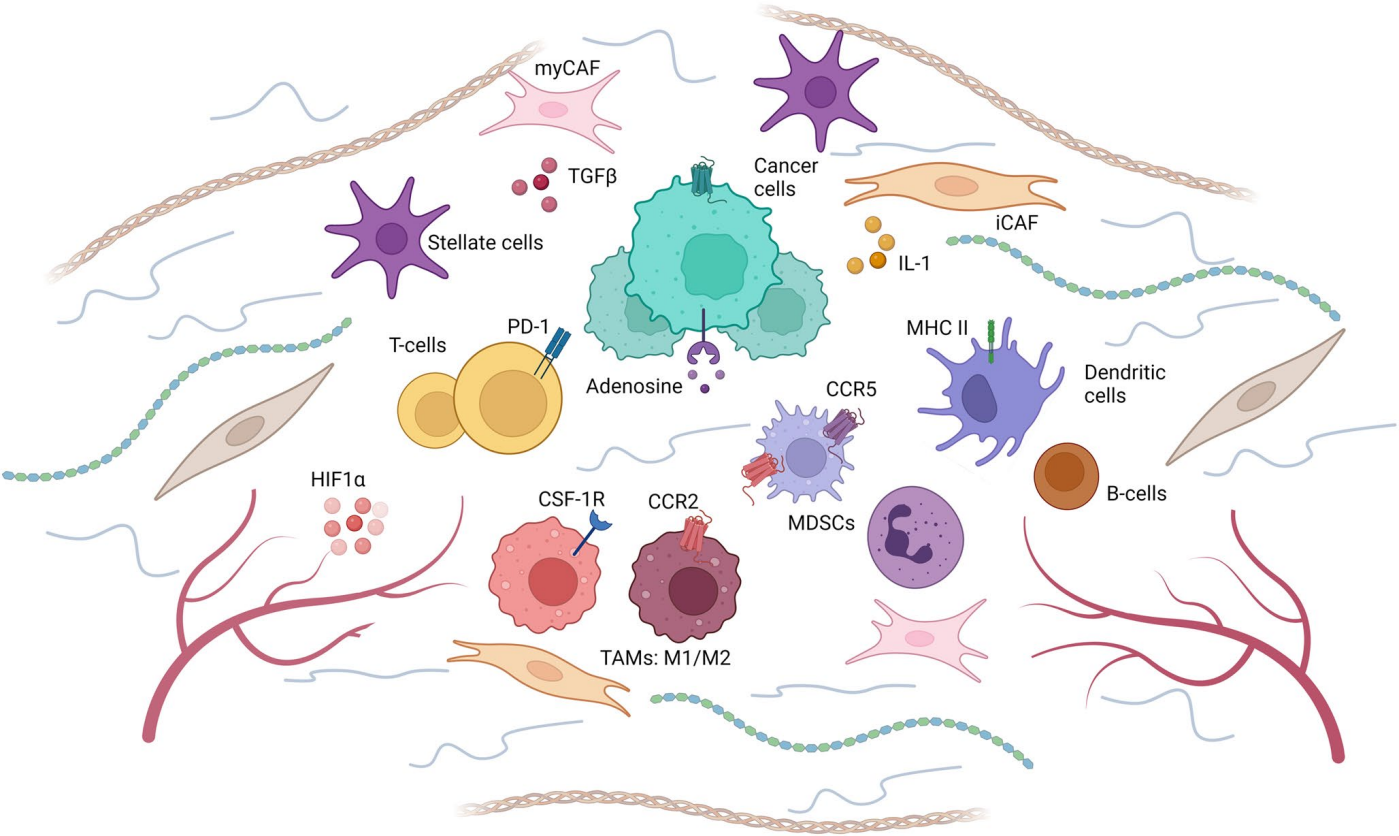
Tumor microenvironment of pancreatic adenocarcinoma. Created with BioRender

The PDAC tumor microenvironment is characterized by a dense desmoplastic stroma that surrounds cancer cells [19, 104]. This stroma is made up of an extensive extracellular matrix (ECM) composed of collagen and hyaluronic acid [105]. Cellular components of the stroma include cancer-associated fibroblasts (CAFs), pancreatic stellate cells (PSCs), infiltrating immune cells, and endothelial cells (Fig. 2). CAFs and PSCs contribute to stromal formation through interactions with both immune and tumor cells [19]. These processes create a high-pressure, hypoxic environment that is characterized by increased immune suppression, altered metabolism, and potentially decreased drug penetration which contribute to the clinical aggressiveness of PDAC [105, 106].

### Stromal Targets

The unique desmoplastic stroma of pancreatic cancer is the result of multicellular interactions in the PDAC TME. In particular, CAFs play key roles in both tumor progression and tumor suppression [107]. Several subtypes of CAFs have been identified in the PDAC TME: inflammatory CAFs, myofibroblastic CAFs, antigen presenting CAFs, and mesenchymal stem cell CAFs [19]. Although they were once presumed to only derive from PSCs, recent research has shown that they make up a small proportion of CAFs [108]. Regardless of the origin, these cells respond to chemokines and contribute to the TME through complex interactions with cancer cells [109].

Multiple studies have been conducted targeting PDAC’s characteristic stroma (Table 5). Prior studies targeting hyaluronic acid in the ECM through the use of PEGPH20 and TGFβ signaling (hypothesized to affect CAF differentiation) through use of NIS739 respectively have not led to improved clinical outcomes in phase III studies [110, 111]. Recently completed trials evaluating the inhibition of connective tissue growth factor (CTGF) using the monoclonal antibody pamrevlumab both in the metastatic and locally advanced setting were reported in abstract form. Despite promising phase II data, pamrevlumab did not demonstrate improvement over chemotherapy alone in the locally advanced or metastatic settings [112, 113].

**Table 5:** PDAC stroma targeting trials

Mechanism	Drug	Phase of study	NCT	Details	Status
Hyaluronidase	PEGPH20	III	NCT02715804: PEGPH20 combined with G/A versus G/A	Primary endpoint OS: PEGPH20 + GA: 11.2 months G/A: 11.5 months[110] (HR = 1.00; 95% CI, 0.80–1.27; *p* = 0.97)	No other active trials
CTGF inhibitor	Pamrevlumab	III	NCT03941093: Pamrevlumab combined with G/A versus G/A in LAPC	Primary endpoint OS: Pamrevlumab + GA: 17.3 months G/A: 18.0 months (HR = 1.08; 95% CI 0.83–1.41, *p* = 0.5487)[112]	No other active trials with pamrevlumab
			NCT04229004: Pamrevlumab combined with G/A versus G/A in 1st /2nd line metastatic PDAC	Model-estimated HR = 1.18, 95% CI 0.88-1.56[113]	
TGFβ inhibitor	NIS739	III	NCT04935359	1st line metastatic PDAC, in combination with G/A vs. G/A alone	Terminated for strategic reasons
Renin-angiotensin inhibitor, possible suppression of TGFβ	Losartan	I	NCT04106856	Neoadjuvant, in combination with hypofractionated RT	Recruiting
		I	NCT05365893	Neoadjuvant, In combination with paricalcitol and hydroxychloroquine	Recruiting
		II	NCT05077800	In combination with FOLFIRINOX and GSK- 3β inhibitor (9-ING-41)	Recruiting
Vitamin D, decreased fibrosis through pancreatic stellate cells	Paricalcitol	II	NCT04524702	In combination with G/A and hydroxychloroquine	Active, not recruiting
		II	NCT03520790	In combination with G/A	Pending results
FAK inhibitor	Defactinib	II	NCT03727880, NCT04331041	Neoadjuvant, in combination with pembrolizumab	Recruiting
		II	NCT04331041	In combination with SBRT	Recruiting
	AMP945	I/II	NCT05355298	In combination with G/A: 6/14 PRs, ORR = 43%[114]	Active, not recruiting
	CT-707	I/II	NCT05580445	In combination with toripalimab and gemcitabine	Recruiting

Pathways currently being explored in ongoing trials targeting the stroma include inhibition of the renin-angiotensin pathway (using losartan), vitamin D receptor pathways, and FAK [114]. Hyaluronic acid is also being targeted through an oncolytic virus strategy (NCT05673811). It is notable that to date, all reported phase III studies of agents targeting these pathways have failed to recapitulate benefits seen in pre-clinical models and early phase trials. Dual roles of CAFs and the TME are likely more complicated than what is currently described in the literature and manipulation of signaling pathways may not yield desired outcomes. However, current studies and biological correlates from patient samples will greatly inform future approaches.

### Immune Targets in TME

Pancreatic cancer has traditionally not been responsive to immune checkpoint blockade unless the tumor is microsatellite unstable which only makes up 1–2% of all PDAC [115]. In the phase II KEYNOTE-158 patients, 22 patients with microsatellite unstable PDAC were enrolled and treated with pembrolizumab in the second line setting, with an ORR of 18%[116]. Despite the low ORR in this trial, high ORRs (> 50%) and prolonged survival benefit with immunotherapy has been reported in real world settings [117, 118]. Given the low percentage of microsatellite unstable PDAC tumors, additional strategies are necessary to promote immune activation in pancreatic cancer. Novel checkpoint blockade agents are being studied in clinical trials, including bispecifics (Table 6).

**Table 6:** Immune-based TME targeting trials

Mechanism	Drug	Phase of study	NCT	Details	Status
CTLA-4 inhibitor	Botensilimab	II	NCT05630183	Randomized phase II, in combination with G/A, 2nd line PDAC	Active, not recruiting
	Other agents in trials: ONC-392 (NCT04140526), XTX101 (NCT04896697)				
PD-L1 inhibitor	IMM2510 (PD-L1 x VEGFR)	I	NCT05972460	Monotherapy	Recruiting
	Ivonescimab (PD-L1 x VEGFR)	II/II	NCT06844422, NCT06491472	In locally advanced PDAC, in combination with either G/A or mFOLFIRINOX	Recruiting
	ATG-101 (PD-L1 × 4-1BB)	I	NCT05490043, NCT04986865	Monotherapy	Recruiting
	ABL503 (PD-L1 × 4-1BB)	I	NCT04762641	Monotherapy	Recruiting
GSK-3β inhibitor	Elraglusib	II	NCT03678883	1- year OS in PDAC: 43.6% survival with elraglusib and G/A compared to 22.5% with G/A[119]	Phase 3 proposed, also in combination in other trials
IL1RAP antibody	Nadunolimab	I/II	NCT03267316	Higher levels of IL1RAP was associated survival of 14.2 months in combination with G/A[120]	Also in trial in combination with FOLFIRINOX (NCT04990037)
CCR2/CCR5 antagonist	BMS-813,160	I/II	NCT03767582	Locally advanced PDAC, in combination with anti-PD-1 ± GVAX	Pending results
CXCR4 antagonist	Motixafortide	II	NCT04543071	In combination with G/A and anti-PD1 (cemiplimab)	Recruiting
IL-1β antagonist	Canakinumab	I	NCT05984602, NCT04581343	Neoadjuvant for PDAC in combination tislelizumab and G/A; in metastatic PDAC, in combination with spartalizumab and G/A	Pending results for both trials
IRAK4 antagonist	CA-4948	I	NCT05685602	2nd line PDAC in combination with G/A	Recruiting
CD40 agonist	sotigalimab	II	NCT03214250	1-year OS: sotigalimab/G/A (48.1%, *p* = 0.062) sotigalimab/nivolumab/G/A (41.3%, *p* = 0.223)[121]	Completed
	mitazalimab	I/II	NCT04888312	In combination with mFOLFIRINOX: ORR = 40% (met primary endpoint of 30%)[122]	Planned phase 3 with mFOLFIRINOX

The immunosuppressive environment of PDAC is partly related to its desmoplastic TME. Cytokines excreted in the TME lead to recruitment of immune suppressive cells such as tumor associated M2 macrophages (TAMs), T-regulatory cells, and myeloid-derived suppressor cells (MDSC) [109]. Characterization of chemokines that contribute to this environment have identified several potential targets for current and future clinical trials. Cytokines and receptors that have been shown to play roles in the PDAC TME include Il-1, CSF-1, CCR2/5, CXCL12/CXCR4, and CD40. Below, we discuss several agents that are aimed are modulating the immune TME of PDAC.

GSK-3β is a serine/threonine kinase that has been shown to be overexpressed in PDAC [123]. In preclinical studies, it has been shown to have several immunomodulatory properties including regulation of PD-1 and LAG-3 expression [124]. Elraglusib is a GSK-3β inhibitor that was recently studied in a randomized phase II trial in metastatic PDAC in the first-line setting. In the trial, elraglusib in combination with G/A met its primary endpoint of 1- year OS, with 43.6% survival rate compared to 22.5% with G/A alone [119]. Interim analysis also showed mOS of 9.3 months with elraglusib versus 7.2 months with chemotherapy and PFS of 5.6 months in the investigational arm versus 4.9 months in the chemotherapy arm. A phase III trial is planned for the future.

IL-1β has been shown in preclinical models to lead to differentiation of inflammatory CAFs and recruitment of immune suppressive cells. Combination with anti-IL-1β and anti-PD1 treatment in a mouse model led to increased CD8^+^ T cells and decreased tumor weight [125]. Canakinumab is an anti-IL-1β antibody that was previously studied in cardiac inflammation and is now being combined with anti-PD-1 on chemotherapy backbone of gemcitabine and nab-paclitaxel (NCT05984602, NCT04581343). Another potential IL-1 therapeutic agent is nadunolimab, an antibody that blocks IL-1α and IL-1β signaling through antibody-dependent cellular cytotoxicity targeting of Interleukin-1 Receptor Accessory Protein (IL1RAP) [120]. In a phase II trial, nadunolimab in combination with G/A in the first-line setting demonstrated overall survival of 13.2 months, a small improvement overall historical data. Higher level of IL1RAP was associated with increased survival of 14.2 months, compared to 10.6 months in lower levels. Further exploration of this pathway with IL1RAP selection for higher levels may yield improved outcomes.

CSF-1 has been shown to induce polarization of macrophages to the M2 phenotype that is tumor promoting [126]. Inhibition of CSF-1 has shown to decrease TAMs and increase PD-L1 in preclinical models of PDAC, although inhibition of this pathway has not been proven to be successful in clinical trials [127]. In one recent phase I study, inhibition of CSF-1R (pexidartinib) and PD-L1 (durvalumab) was shown to impair dendritic cells through FLT3 antagonism, possibly explaining the limited clinical efficacy seen [128]. CCR2 has also been implicated in TAM trafficking. In a prior phase Ib trial using the CCR2 inhibitor PF-04136309, there was no clear clinical benefit when combined with G/A and concerns about toxicity [129]. This was despite a promising phase Ib trial combining PF-04136309 with FOLFIRINOX, which showed an ORR of 49% (*n* = 33) compared to 0% (*n* = 5) in the study’s FOLFIRINOX group, although the study was not randomized [130]. However, a dual CCR2/5 antagonist BMS-813,160 has completed accrual in several studies and results are pending.

Inhibition of CXCR4/CXCL12 has also been shown to increase T-cells in the TME of PDAC and improve responsiveness to PD-1 blockade [131, 132]. A phase II trial evaluated the combination of pembrolizumab with CXCR4 inhibition (motixafortide) and NALIRI after progression on prior chemotherapy. The ORR was 21.1% (13.2% confirmed), mPFS of 3.8 months, and mOS of 6.6 months. Another phase II trial combining motixafortide with anti-PD1 (cemiplimab) and G/A in the first line setting is currently in process. CD40 is another target that has been shown to promote T-cell activity and decrease tumor stroma through dendritic cell activity within the PDAC TME [133]. In a phase II study, CD40 agonist sotigalimab did not improve overall survival at 1 year when added to G/A as a single agent or with nivolumab when compared to control: sotigalimab/chemo (48.1%, *P* = 0.062, *n* = 36) or sotigalimab/nivolumab/chemo (41.3%, *P* = 0.223, *n* = 35) [121]. However, an alternative CD40 agonist, mitazalimab was combined with FOLFIRINOX in a phase II trial with an ORR of 40%, meeting the primary endpoint [122]. A larger phase III trial is planned.

### Novel Antibodies and Bispecifics

In addition to direct immune modulation, there are several antibody-drug conjugates (ADC) and bi-specific antibodies that are being tested in PDAC. Traditional targets such as PD-L1 and CTLA-4 are described in Table 7. In addition to these two targets, claudin 18.2 has emerged as an important receptor for drug targeting, with promising results already demonstrated for gastric cancer (Table 8) [134–136]. Zolbetuximab, which was approved in gastric cancer as a monoclonal antibody is also under investigation in PDAC (NCT03816163). IBI343 is an ADC targeting Claudin 18.2 with a topoisomerase payload that demonstrated 40% ORR in phase I trial in refractory PDAC patients [137]. IBI389 is a Claudin 18.2 and CD3 bispecific that was reported to have an ORR of 30.4% in refractory PDAC [138]. PT886 is another Claudin 18.2 bispecific in combination with CD47, which is overexpressed by tumor cells and regulates phagocytosis [139, 140].

**Table 7:** Bispecifics, antibody drug conjugates, and CAR-T in development

Mechanism	Drug	Phase	NCT	Details	Status
Bispecific Antibodies	AK104 (PD-1/CTLA-4)	I	NCT05859750	In combination with chemotherapy in 1st line setting	Recruiting
	EGFR FPBMC (EGFR/CD3)	I	NCT06479239	Recruiting, Monotherapy, bispecific is added ex vivo before infusion	Recruiting
	AGEN1423 (Anti-CD73/TGFβ-Trap)	II	NCT05632328	In combination with botensilimab ± G/A	Recruiting
	ARB202 (CDH17/CD3)	I	NCT05411133	Monotherapy and in combination with atezolizumab	Recruiting
	NI-1801 (mesothelin/CD47)	I	NCT05403554	Monotherapy and in combination with pembrolizumab or paclitaxel	Recruiting
Antibody Drug Conjugates	MRG004A (Tissue Factor with MMAE)	I/II	NCT04843709	In PDAC: ORR was 33.3% (4/12) and DCR was 83.3% (10/12). PR seen in 4/5 patients with tissue factor overexpression [138]	Recruiting
	LY4101174 (Nectin-4 ADC with Topoisomerase I exatecan)	I	NCT06238479	Monotherapy	Recruiting
	LCB84 (TROP2 ADC with MMAE)	I	NCT05941507	Monotherapy and in combination with anti-PD1	Recruiting
	MGC028 (ADAM9 ADC with Topoisomerase I SYNtecan E™)	I	NCT06723236	Monotherapy	Recruiting
	M9140 (CEACAM5 ADC with Topoisomerase 1 payload exatecan)	I	NCT05464030	Monotherapy and planned combination groups	Recruiting
	EBC-129 (CEACAM5/6 ADC with MMAE)	I	NCT05701527	Monotherapy and in combination with pembrolizumab	Recruiting
	ABBV-400 (cMET ADC with topoisomerase 1)	I	NCT06084481	Monotherapy	Recruiting
CAR-T	Mesothelin targets: • A2B694 (Gated Mesothelin CAR-T) with HLA-A*02:01 and tumor expressing mesothelin: NCT06051695 • huCART-meso: NCT03323944 • CHT102: NCT06760364 • UCMYM802 (circular mRNA encoding anti-mesothelin CAR-T): NCT06256055 Mesothelin/GPC3/GUCY2C CAR-T: NCT05779917				
	CEA targets: • A2B530 (Gated CEA CAR-T) with HLA-A*02:01 and tumor expressing CEA: NCT05736731 NCT06010862, NCT06126406, NCT06006390, NCT05415475, NCT06821048, NCT05538195				
	EpCAM target: IMC001 (NCT05028933) CD70 target: CTX131 (allogenic CAR-T modified with CRISPR-Cas9 ex vivo) NCT05795595 MUC1-C target: NCT05239143 B7-H3 target: NCT06158139

**Table 8:** Claudin 18.2 targeting trials

Mechanism	Drug	Phase	NCT	Details	Status
Antibody	Zolbetuximab (Monoclonal antibody against Claudin 18.2)	Randomized phase II	NCT03816163	In combination with G/A vs. G/A alone in 1st line setting	Accrued, pending results
	Other antibodies: AB011 (NCT04400383), TST001 (NCT04495296), ASKB589 (NCT04632108), ML108 (NCT04894825), MIL93 (NCT04671875)				
Bispecific Antibody	IBI389 (Claudin 18.2/CD3 Bispecific Antibody)	I	NCT05164458	In PDAC, ORR of 30.4% [138]	Recruiting, as single agent in combination with sintilimab
	ASP2138 (Claudin 18.2/CD3 Bispecific Antibody)	I	NCT05365581	Metastatic/advanced GI cancers, including PDAC expressing Claudin 18.2	Recruiting, monotherapy and in combination with mFOLFIRINOX, 1st line setting cohort for PDAC
	AZD5863 (Claudin 18.2/CD3 Bispecific Antibody)	I/II	NCT06005493	Metastatic/advanced GI cancers, including PDAC expressing Claudin 18.2	Recruiting
	PT886 (Claudin 18.2/CD47 Bispecific Antibody)	I	NCT05482893	Metastatic/advanced GI cancers, including PDAC expressing Claudin 18.2	Recruiting
	Other bispecific antibodies: • Q-1802 (Claudin 18.2/PD-L1 Bispecific Antibody): NCT04856150 • SG1906 (Claudin 18.2/CD47 Bispecific Antibody): NCT05857332 • PM1032 (Claudin 18.2/4-1BB Bispecific Antibody): NCT05839106				
Antibody-drug Conjugates	IBI343 (Claudin 18.2 ADC with topoisomerase I/exatecan)	I	NCT05458219	In PDAC (*n* = 10), ORR of 40% [137]	Monotherapy reported and recruiting, combination treatment planned with G/A in PDAC (NCT06770439)
	EO-3021 (Claudin 18.2 ADC with MMAE)	I	NCT05980416	In gastric cancer, ORR of 42.8% seen with single agent [134]	Recruiting
	LM-302 (Claudin 18.2 ADC with MMAE)	I/II	NCT05934331	In gastric cancer, ORR of 30.6% seen with single agent [135]	Recruiting, in combination with toripalimab
	AZD0901 (Claudin 18.2 ADC with MMAE)	II	NCT06219941	In gastric cancer, ORR 29% seen with single agent [136]	Recruiting, monotherapy and in combination with chemotherapy in 1st line setting
	Other ADCs targeting Claudin 18.2: • SKB315 (Claudin 18.2 ADC with topoisomerase I): NCT05367635 • RC118 (Claudin 18.2 ADC with MMAE) as single agent and with toripalimab: NCT06038396, NCT05205850 • TORL-2-307 (Claudin 18.2 ADC): NCT05156866				
CAR-T	CT041	I	NCT03874897 NCT04581473	ORR and DCR were 16.7% and 70.8%. mPFS: 3.3 months (95% CI, 1.8 to 6.2), mOS: 10.0 months (95% CI, 5.5 to 17.6) [141]	Ongoing study in metastatic and in adjuvant setting after chemotherapy (NCT05911217)
	Other Claudin 18.2 targeting CAR-T cells: • IBI345: NCT05199519 • LB1908: NCT05539430 • AZD6422: NCT05981235 • KD-496 (NKGD/Claudin 18.2 CAR-T): NCT05583201 • CLDN18.2/PD-L1 dual-targeting CAR-T: NCT06084286 • CT048: NCT06084286 • IMC002: NCT05946226, NCT05837299				

Additional targets for ADC include mesothelin, c-MET, MUC1, and CEACAM5/6 that have been conjugated to various payloads (Table 7). Of interest, an ADC targeting tissue factor and conjugated to MMAE, MRG004A, recently demonstrated 4/5 PRs in PDAC in a phase I/II trial [142]. Similarly, bispecific antibodies have a variety of targets such as VEGFA, 4-1BB, and DLL3. Given the variety of targets available, identification of those that have the highest efficacy as well as those that minimize off-target effects (such as cytokine release syndrome with ADCs) will be key. In addition, combination strategies with chemotherapy or checkpoint blockade may also improve benefit.

### Vaccines and Cellular Therapies

The success of vaccines in PDAC and cancer treatment in general has been limited. Barriers to successful treatments include identifying immunogenic antigens, designing appropriate delivery systems, and overcoming tumor-mediated immunosuppression [143]. Some of the first vaccines targeting pancreatic cancer were based on GM-CSF (GVAX) [144]. GVAX is an allogenic vaccine created from transfecting two primary human pancreatic cells lines to express GM-CSF. GM-CSF was shown in preclinical models to elicit systemic immune responses through its effects on antigen-presenting cells and T-cell activation [145]. In single arm studies, GVAX showed promising activity, especially in combination with other agents such as checkpoint blockade [146, 147]. However, in randomized phase II trials in the metastatic setting, it has not been associated with improved outcomes [148, 149]. Whether there may be beneficial results in the neoadjuvant/adjuvant setting with GVAX treatment is currently being evaluated, with early results showing increased intratumoral cytotoxic T-cells when combined with PD-1 blockade and CD137 agonist [150] (NCT02451982).

The use of mRNA vaccines in COVID-19 pandemic has generated increasing interest in using mRNA vaccines in cancer [151]. Several vaccines are now in trials focusing on utilizing mRNA vaccines to target mutant *KRAS* as well as personalized neoantigens. In a recent phase I trial, 16 patients were treated with a personalized mRNA neoantigen vaccine after pancreatic cancer resection in combination with atezolizumab and mFOLFIRINOX [152]. Post-vaccination neoantigen-specific CD8 + T cells increased in 50% of patients (*n* = 8) from undetectable levels to a median of 2.9%. At a median follow-up of 3.2 years, responders had longer recurrence free survival compared to non-responders (median not reached vs. 13.4 months, HR 0.08, 95% CI 0.01–0.5, *P* = 0.007) [153]. This individualized approach will be tested in a large, randomized Phase II trial (NCT05968326). Vaccines utilizing other strategies, including dendritic cell vaccines and KRAS targeting vaccines are also included in Table 9. A recent dendritic cell vaccine reported a 2-year recurrence free survival of 64% among 28 patients [154]. Similarly, a KRAS vaccine study in patients with positive tumor markers demonstrated early proof of concept with median recurrence-free survival of 15.3 months among 20 patients [155].

**Table 9:** Vaccines in pancreatic cancer

Mechanism	Regimen	Phase of study	NCT	Details	Status
GVAX	GVAX-based vaccine	II	NCT02451982	In neoadjuvant and adjuvant setting, multiple cohorts including with nivolumab and urelumab	Recruiting
	GVAX or KRAS vaccine	I/II	NCT06782932	GVAX or KRAS combine with balstilimab and AGEN2372 (CD173 agonist)	Not yet recruiting
Personalized vaccines	Autogene cevumeran (Personalized mRNA neoantigen vaccine)	Randomized phase II	NCT05968326	Adjuvant PDAC; In phase I portion, patients with vaccine induced T-cells had increased RFS versus those without (NE verus 13.4 months, *p* = 0.007) [153]	Randomized phase II recruiting: Vaccine in combination with anti-PD-L1 (atezolizumab) and mFOLFIRINOX vs. mFOLFIRINOX alone
	Others in clinical trials: • Neoantigen long peptide vaccine with poly-ICLC in neoadjuvant/adjuvant setting (NCT05111353) • Personalized peptide vaccine with imiquimod/sotigalimab/pembrolizumab in metastatic setting (NCT02600949)				
Dendritic based vaccines	Autologous DCs pulsed with allogeneic mesothelioma tumor cell lysate	I/II	EudraCT 2018-003222-92	Vaccinated after chemotherapy in adjuvant setting; Primary endpoint 2-year RFS: 64% [154]	Vaccine being used in additional trial (NCT05650918)
	Others in clinical trials: • Dendritic vaccine targeting tumor lysate and mRNA in the adjuvant setting (NCT04157127) • Dendritic vaccine with IL-15 targeting WT-1 in metastatic setting (NCT05964361) • Dendritic vaccine targeted against allogenic mesothelioma tumor lysate with mitazalimab in metastatic setting (NCT05650918)				
KRAS based vaccines	ELI-002: a lipid-conjugated immune-stimulatory oligonucleotide with lipid-conjugated peptide-based antigens against KRAS/NRAS	I/II	NCT05726864	Adjuvant PDAC in patients with positive tumor markers: mRFS in the PDAC subgroup (*n* = 20, 15.3 months) [155]	Randomized phase II portion results pending
	Others in clinical trials: • KISIMA platform peptide vaccine against KRAS G12D/V with anti-PD1 in locally advanced, adjuvant, and metastatic cohorts (NCT05846516) • Pooled mutant-KRAS long peptide vaccine with nivolumab/ipilimumab in adjuvant PDAC (NCT04117087) • Dendritic cell vaccine targeting mutant KRAS in adjuvant PDAC (NCT03592888) • TG01/QS-21 (RAS-neoantigen peptide vaccine) ± balstilimab in adjuvant PDAC with positive ctDNA (NCT05638698) • KRAS long peptide vaccine with poly-ICLC in patients at high risk for developing PDAC (NCT05013216)				
Other mechanisms	Ad5.F35-hGCC-PADRE (adenovirus-based vaccine targeting guanylyl cyclase C)	II	NCT04111172	Adjuvant therapy in PDAC	Active, not recruiting
	TGFβ−15 peptide vaccine	I	NCT05721846	In combination with nivolumab and ipilimumab	Recruiting

Similar to other solid tumors, adoptive T cell therapy is also being developed for pancreatic cancer. In a recent report, autologous T cells of a treatment-refractory pancreatic cancer patient were engineered to target KRAS^G12D^ when presented on HLA-C*08:02 ligand [156]. In this single patient case report, significant clinical benefit was seen, with 72% partial response that was sustained at 6 months. Although promising, whether such an approach can be adopted on a large-scale basis and outside of this specific HLA type are important considerations. Other engineered T-cells against KRAS mutations trials are also ongoing (Table 10).

**Table 10:** Autologous T-cell products

	Product	Phase	NCT	Details	Status
Autologous T cells	FH-TCR-T_MSLN_ (Mesothelin-Specific Autologous T-Cells)	I	NCT04809766	Metastatic PDAC expressing mesothelin	Active, not recruiting
	NT-175 (HLA-A*02:01-restricted TCR, targeting TP53 R175H)	I	NCT05877599	HLA-A*02:01 subjects with advanced PDAC and TP53 R175H mutation, addition of IL-2 post-infusion	Recruiting
	HLA-A*11:01 TCR T-cells targeting TP53 R248Q	I	NCT06619886	HLA-A*11:01 subjects with advanced PDAC and TP53 R248Q mutation	Not yet recruiting
	NT-112 (HLA-C*08:02-restricted TCR, targeting KRAS G12D)	I	NCT06218914	HLA-C*08:02 subjects with advanced PDAC and KRAS G12D mutation, addition of IL-2 post-infusion	Recruiting
	Murine derived TCR against KRAS G12D	I	NCT03745326	HLA-A*11:01 subjects with KRAS G12D	Recruiting
	TCR1020-CD8 T cell targeting KRAS G12V	I	NCT06707896	HLA-A*11:01 subjects with KRAS G12V	Not yet recruiting
	AFNT-211 targeting KRAS G12V	I	NCT06105021	HLA-A*11:01 subjects with KRAS G12V	Recruiting
	Murine derived TCR against KRAS G12V	I	NCT03190941	HLA-A*11:01 subjects with KRAS G12D	Recruiting
	FH-A11KRASG12V-TCR targeting KRAS G12V	I	NCT06043713	HLA-A*11 subjects with KRAS G12V	Active, not recruiting
	IX001 TCR against KRAS mutations	I	NCT06487377	KRAS-G12V/D mutations and HLA-A*11, C*01:02, or C*08:02, Addition of post-infusion IL-2	Recruiting

Chimeric antigen receptor T cells (CAR-T) are also an emerging area of study after successes in hematological malignancies [157]. Similar to ADCs, development of CAR-Ts targeting markers such as mesothelin, claudin 18.2, and CEA are pending, with early results reported for CT041, a Claudin specific CAR-T [141, 158] (Tables 7 and 8). Strategies to minimize off-target toxicity will be important. A CAR-T that uses HLA-A*02 to discriminate between tumor and normal cells to limit toxicity is currently in phase I trials (NCT06051695, NCT05736731).

### Metabolic Modulators

Both the hypoxic environment of the PDAC stroma and mutations in *KRAS* have been shown to cause changes in several metabolic pathways that are of clinical interest. Increased dependence on lysosome scavenging pathways, increased glycolysis, and reliance on non-canonical glutamine metabolism have been shown with PDAC [159]. Despite these key differences, modulation of metabolism has been challenging in clinical practice. A recent phase III trial utilizing demvistat, a mitochondrial metabolism modulator targeting pyruvate dehydrogenase (PDH) and alpha-ketoglutarate dehydrogenase (α-KGDH) involved in the TCA cycle in combination with FOLFIRINOX chemotherapy failed to show an improvement in survival [160].

Alterations in autophagy dependence have been shown in PDAC in several preclinical studies [161–163]. Increased autophagy has been associated with decreased MHC I expression and subsequent immune suppression [162]. However, targeting of autophagy has had limited success clinically in part due to lack of drugs targeting autophagy. Hydroxychloroquine has been used in prior trials, but it is not a pure autophagy inhibitor. In two different randomized phase II trials utilizing hydroxychloroquine in combination with G/A in either the preoperative or the advanced disease setting, the addition of hydroxychloroquine did not show increased survival, although there was some increase in pathological responses at time or surgery and overall response rate [164, 165]. Several upcoming trials are combining hydroxychloroquine with additional therapies (Table 11). A novel inhibitor of autophagy, DCC-3116, that acts through ULK1/2 kinases has also recently entered phase I/II clinical trials (NCT04892017).

**Table 11:** Metabolism targeting strategies

Mechanism	Drug	Phase	NCT	Details	Status
Autophagy inhibitor	Hydroxychloroquine	I	NCT05733000	In combination with devimistat and chemotherapy	Recruiting
		I/II	NCT04911816	Neoadjuvant PDAC, In combination with mFOLFIRINOX	Recruiting
		I	NCT03825289	In combination with MEK inhibitor (trametinib)	Recruiting
		I	NCT04132505	In combination with MEK inhibitor (binimetinib)	Active, not recruiting
ULK1/2 kinase	DCC-3116	I	NCT04892017	PDAC with RAS, RAF, or NF1 mutation, monotherapy and in combination with MEK inhibitor (trametinib)	Recruiting
CD73 inhibitor	AB680/Quemliclustat	III	NCT06608927	In phase I trial, ORR was 41% in treatment-naïve metastatic patients with mOS of 21 months [166]	Recruiting phase III randomized clinical trial with AB680 with G/A versus G/A alone
		I/II	NCT05688215	Neoadjuvant PDAC: In combination with anti-PD1 zimberelimab and mFOLFIRINOX	Recruiting
		I/II	NCT06048484	In combination with SBRT, zimberelimab, ± etrumadenant (inhibitor of adenosine 2 A/B receptors)	Recruiting
PRMT5 inhibitor	AMG193	I/II	NCT05094336, NCT06360354	In PDAC cohort (*n* = 16), 12.5% ORR and 43.8% DCR [167]	Recruiting for monotherapy and chemotherapy combination cohorts for mFOLFIRINOX and G/A
	MRTX1719	I/II	NCT05245500	In PDAC (*n* = 30), 10% ORR, 73% DCR [168]	Recruiting
	TNG462	I/II	NCT05732831	Monotherapy for MTAP deleted tumors	Recruiting
MAT2A inhibitor	IDE397	I	NCT04794699	Monotherapy for MTAP deleted tumors and in combination with AMG 193	Recruiting
	ISM3412	I	NCT06414460	Monotherapy for MTAP deleted tumors	Recruiting
	S095035	I	NCT06188702	Monotherapy for MTAP deleted tumors	Recruiting

PDAC tumors also demonstrate increased CD73 expression, an ectonucleotidase that catalyzes the formation of extracellular adenosine. Preclinical studies have demonstrated that increased CD73 correlates with decreased CD4^+^ and CD8^+^ T cells in tumor samples [169]. Adenosine has been shown to promote immune suppression through decreases in T-cell proliferation and activation [170, 171]. In a phase I/Ib trial utilizing quemliclustat, CD73 antibody in combination G/A, ORR was 41% in treatment-naïve metastatic patients with mOS of 21 months [166]. A phase III double-blinded trial evaluating the addition of quemliclustat to G/A is currently in progress (NCT06608927). TTX-030, an anti-CD39 antibody, is another adenosine targeting agent that demonstrated a promising mOS of 19.1 months when added to G/A with or without anti-PD1. A randomized phase II trial is planned [172].

An additional pathway of interest is alterations in the methionine salvage pathway, through the loss of methylthioadenosine phosphorylase (MTAP). MTAP plays a key role in the methionine salvage pathway by catalyzing the breakdown of 2-methylthioadenosine (MTA). Given its proximity to *CDKN2A* on chromosome 9, *MTAP* is frequently co-deleted in PDAC. Overall, approximately 20–30% of PDAC have MTAP loss [173, 174]. MTAP loss leads to accumulation of methylthioadenosine which leads to inhibition of protein arginine methyltransferase 5 (PRMT5) [175]. Further inhibition of PRMT5 showed synthetic lethality in certain preclinical models of PDAC [176, 177]. An additional target of this pathway is MAT2A, which acts upstream of PRMT5 and catalyzes formation of its substrate S-adenosylmethionine (SAM). Several PRMT5 and MAT2A inhibitors are in clinical trials, including in combination together, with early results showing disease control rate of 43.8% and 73% [167, 168, 178]. Future trials with PRMT5 and KRAS inhibitors in combination are also planned [179].

## Areas of Clinical Opportunities

In addition to novel systemic targets, significant clinical questions remain on how to utilize current treatment modalities and sequencing of treatments. Although neoadjuvant treatment appears to be useful for borderline resectable PDAC, there is still ongoing debate regarding upfront surgery versus neoadjuvant chemotherapy with or without radiation in resectable disease. The optimal regimen and the role of radiation are still under study. In resectable patients, several randomized chemotherapy alone studies are open including the PREOPANC-3 and ALLIANCE-A021806 study. The PREOPANC-2 trial is currently in process evaluating the efficacy of neoadjuvant FOLFIRINOX compared to neoadjuvant gemcitabine and CRT in both resectable and borderline resectable patients [180]. In addition to radiation in the neoadjuvant setting, the overall benefit of radiation in PDAC is still debated. Although radiation offers improved local control, it has not definitely shown improvements in overall survival [181]. However, time off chemotherapy may be a meaningful clinical endpoint for patients. Furthermore, as ongoing clinical trials improve on systemic therapies, local control may have a greater role in the future.

In the unresectable setting, the optimal sequence of available chemotherapy combinations is being tested. There has been no randomized trial comparing FOLFIRINOX G/A in the frontline setting though data from the NAPOLI-3 trial clearly show a modest but significant survival benefit from NALIRIFOX over gem/nab-p in metastatic patients [12]. In addition, whether utilizing both regimens at certain points before progression is an active area of investigation. On a biological level, utilizing alternating regimens potentially targets increased number of molecular subtypes of PDAC. In a recent trial presented at ASCO 2022, the phase II SEQUENCE trial compared gemcitabine/nab-paclitaxel followed by FOLFOX with gemcitabine/nab-paclitaxel alone. This showed a 12-month OS of 55.3% in the G/A/FOLFOX arm compared with 35.4% which met the primary endpoint. Whether this regimen will show benefit for those who can tolerate FOLFIRINOX is still an open question.

Although most non-resectable patients with PDAC ultimately progress on treatment, a small minority experience prolonged stability and the best maintenance strategy in these patients is unclear given the side effects of continued chemotherapy. Decreased chemotherapy intensity (capecitabine/5-FU or FOLFIRI for FOLFIRINOX, gemcitabine as single agent) and less frequent dosing are currently 2B recommendations as part of NCCN guidelines. As previously mentioned, the POLO trial utilized PARP inhibitors in those with germline *BRCA1/2* mutations after treatment with chemotherapy. In the phase II PANOPTIMOX-PRODIGE 35 trial, 5-FU was tested as maintenance therapy after a set period of FOLFIRINOX compared to upfront FOLFIRINOX for 6 months and FOLFIRI alternating with gemcitabine [182]. 5-FU as maintenance therapy showed similar PFS survival to FOLFIRINOX (42.9% versus 47.1%), although the group that received maintenance 5-FU ultimately had increased cumulative doses of oxaliplatin and resulting neurotoxicity. Whether targeted therapies or immune therapies have a role in minimizing toxicity of chemotherapy are important questions being addressed in maintenance trials.

## Conclusions and Future Directions

PDAC remains a difficult cancer to treat and improvements in survival achieved in the last decades are limited, although meaningful for a subset of patients. Currently, the mainstay of treatment in metastatic patients remains multi-agent chemotherapy with limited roles for targeted agents and immunotherapy. However, recent promising results in targeting KRAS are clinically promising, and if proven, can lead to profound changes in therapeutics. In addition, KRAS combinations with immunotherapy or targets of the tumor microenvironment can perhaps reveal new synergistic combinations and supplement or supplant current chemotherapy approaches. KRAS inhibition may help reverse PDAC’s fibrotic stroma and lead to improved drug delivery of other potential therapeutics. Similar to other cancers, novel antibodies and T-cell therapies are being developed and may show clinical efficacy. Given the multitude of therapeutic approaches, the challenge in the future will be to define rational combinations and the appropriate clinical context.

## Key References

- Halbrook, C.J., Lyssiotis, C.A., Pasca di Magliano, M. & Maitra, A. Pancreatic cancer: Advances and challenges. *Cell***186**, 1729-1754 (2023).
  - Importance reference providing overview of translational advances
- Wainberg, Z.A.*, et al.* NALIRIFOX versus nab-paclitaxel and gemcitabine in treatment-naive patients with metastatic pancreatic ductal adenocarcinoma (NAPOLI 3): a randomised, open-label, phase 3 trial. *The Lancet*.
  - Important reference establishing NALIRIFOX as superior to gemcitabine/*nab-*paclitaxel
- Jiang, J.*, et al.* Translational and Therapeutic Evaluation of RAS-GTP Inhibition by RMC-6236 in RAS-Driven Cancers.*Cancer Discov* **14**, 994-1017 (2024).
  - Outstanding important reference given its background into *in vitro *and clinical efficacy of a pan-RAS inhibitor
- Ho, W.J. & Jaffee, E.M. Macrophage-Targeting by CSF1/1R Blockade in Pancreatic Cancers. *Cancer Res* **81**, 6071-6073 (2021).
  - Important reference providing overview of CSF1 treatments previously studied in pancreatic cancer
- Sethna, Z.*, et al.* RNA neoantigen vaccines prime long-lived CD8+ T cells in pancreatic cancer. *Nature* (2025).
  - Outstanding important references describing use of adjuvant vaccines in pancreatic cancer and longterm clinical outcomes

## Data Availability

No datasets were generated or analysed during the current study.
